# Clozapine-induced interstitial nephritis - a rare but important complication: a case report

**DOI:** 10.4076/1752-1947-3-8574

**Published:** 2009-08-27

**Authors:** Robert Hunter, Tracey Gaughan, Filippo Queirazza, Dina McMillan, Susan Shankie

**Affiliations:** 1 Gartnavel Royal Hospital, Glasgow G12 0XH, Scotland, UK; 2 Psychiatric Research Institute for Neuroscience in Glasgow, West Medical Building, University of Glasgow, Glasgow G12 8QQ, Scotland, UK

## Abstract

**Introduction:**

Given the limited range of effective drug treatments for patients with schizophrenia, increasing numbers of patients, often termed 'treatment-resistant' are prescribed clozapine. While the induction of neutropenia or agranulocytosis by clozapine is well appreciated, other rare potentially fatal adverse reactions may also occur including acute interstitial nephritis as reported in this case.

**Case presentation:**

A 57-year-old Caucasian woman with treatment-resistant chronic schizophrenia developed acute renal failure following initiation of treatment with clozapine. The adverse reaction occurred after only four doses of the drug had been administered (titrated from 12.5 to 25 mg per day). After clozapine had been withdrawn, the patient's renal function returned to normal with no other changes to medication. The patient had been exposed to clozapine about 4 years previously when she had developed a similar reaction.

**Conclusion:**

Renal reactions to clozapine are extremely rare but, if not recognized promptly, may prove fatal. Psychiatrists need to be aware of this possible complication when clozapine is initiated.

## Introduction

The dibenzodiazepine derivative, clozapine, is a so-called 'atypical' or second-generation antipsychotic that is widely regarded as one of the more effective drug treatments for schizophrenia. It can cause serious adverse effects on the blood, including a potentially fatal neutropenia (around 2.7% of patients treated with clozapine), and its use is therefore usually limited to treatment-resistant cases of schizophrenia. Although a number of uncommon adverse effects are recognised to occur with clozapine, including hepatitis, pancreatitis, vasculitis and pneumonia [9], renal adverse events are less well recognised. We report a case of acute renal failure, assumed to be due to interstitial nephritis that developed in a 57-year-old Caucasian woman with treatment-resistant chronic schizophrenia following initiation of treatment with clozapine. Although renal complications of clozapine use are very rare, it is essential that psychiatrists using clozapine are aware of the potential for this adverse reaction in order to instigate prompt treatment if necessary.

## Case presentation

The adverse reaction to clozapine occurred in a 57-year-old married Caucasian woman with a long history of treatment-resistant chronic schizophrenia (ICD10 F20) who has required continuing care in hospital for many years, due to the severity of her condition. She first developed schizophrenia at around the age of 18 and has received antipsychotic medication for almost 40 years. There is a family history of schizophrenia with her son and a maternal cousin both having the condition. Despite the recognized advantages of treating many patients with schizophrenia in community settings, her symptoms - both positive and negative - have been resistant to a range of treatments and as a consequence she has been in hospital since 1985. Periodically, the patient can also exhibit challenging behaviour that requires skilled nursing support; on occasion, she has required nursing in the intensive psychiatric care unit rather than the rehabilitation ward. The patient has been prescribed many different antipsychotics over the 40 year duration of her illness, including second-generation (atypical) antipsychotic medication, with unfortunately no benefit to symptoms or functioning. A trial of clozapine in June 2003 was discontinued about a month later following development of acute renal failure, when she required transfer to the general hospital unit. At that time, the renal failure was attributed to clozapine by internal medicine specialists, although the patient was also taking lithium at this time, and developed secondary lithium toxicity with a peak lithium of 2.2 mmol/L before all medication was discontinued.

In November 2007, after a multidisciplinary case review, it was decided that the patient might benefit from a retrial of clozapine, especially given her failure to benefit from all atypical antipsychotics available in the UK. This was considered a reasonable strategy given that the adverse renal effects seen previously when clozapine had been introduced, were thought to have been caused, or exacerbated by, lithium carbonate. Given the severe and refractory nature of the patient's condition, and taking into account the circumstances of the previous trial of clozapine, there appeared compelling grounds for a reintroduction of clozapine. The patient's concomitant medication at the time of the retrial was olanzapine 10 mg and levomepromazine 75 mg at night and sodium valproate 1100 mg per day; for acute exacerbations of psychosis, haloperidol 5 mg IM was available. This combination of medication had been utilized for several months before the clozapine retrial, without evidence of adverse effects. Before the treatment trial of clozapine, treatment with levomepromazine was discontinued. Haematology and biochemistry blood results were normal when checked 3 days before clozapine was initiated.

Clozapine was started with a dose of 12.5 mg at night. On day 2 of clozapine treatment (12.5 mg in the morning and at night), the patient complained of feeling generally unwell and on examination was tachycardic (115 beats per minute) and pyrexial (37.5°C). On day 3 (12.5 mg clozapine in the morning and at night), the patient remained tachycardic and pyrexial (38.0°C). A urine dipstick test showed a trace of protein and the presence of red blood cells (RBCs) and the patient was presumed to have a urinary tract infection and was commenced on trimethoprim, after a urine (MSSU) sample had been sent for bacteriology. On day 4, the patient's pyrexia and tachycardia persisted, and clozapine was stopped; a total of five doses of clozapine had been given. Blood biochemistry revealed a markedly elevated C-reactive protein (CRP) of 197 mg/L; differential white cell count: lymphocytes 1.04 (1.3-3.5 × 10^9^) and neutrophils 8.2 (2.0-7.5 × 10^9^). All other results were normal including creatinine (87 μmol/L). Midstream specimen of urine showed scanty pus cells and organisms, but no RBCs, and no growth. The following day, trimethoprim was discontinued and amoxicillin commenced for a suspected chest infection. The raised temperature settled over the next few days, although the patient remained tachycardic. Blood biochemistry on day 8 showed CRP 138 mg/L, creatinine 126 μmol/L, and an estimated glomerular filtration rate (eGFR) of 40 mL/min/1.73 m^2^. Biochemistry results on day 9 were creatinine 106 μmol/L, eGFR 50 mL/min/1.73 m^2^, and erythrocyte sedimentation rate (ESR) 70 mm/hour. Amoxicillin was discontinued after 5 days. Renal function returned to normal over the next few days.

## Discussion

Acute renal failure has been reported only rarely in association with clozapine therapy [1]-[5]. Out of a total of over 26,000 suspected adverse reactions to clozapine, the UK Medicines and Healthcare products Regulatory Agency (MHRA) has received only 10 reports of acute interstitial nephritis and 31 reports of acute renal failure (January 2009) [6]. In some of the cases [1,4], the diagnosis had been confirmed by biopsy showing that the renal failure had resulted from acute interstitial nephritis. As psychiatrists and other clinicians will appreciate, it is usually not possible to confirm diagnosis by biopsy in most patients with behavioural disturbance due to acute psychosis, despite the recognition that this would normally be clinically desirable. Acute interstitial nephritis is commonly caused by a drug hypersensitivity reaction and, in this patient and other case reports, there was a close temporal relationship between the start of clozapine treatment and the development of interstitial nephritis. In this case, the patient had been previously exposed to clozapine and the rapid onset of the reaction after just five doses of clozapine is consistent with a drug hypersensitivity reaction. Following the withdrawal of clozapine, the patient's renal function returned to normal with no other changes to medication.

Treatment resistance in schizophrenia has been estimated to be around 30% [7], and increasing numbers of patients are being prescribed clozapine; for example, in the Scottish Schizophrenia Outcomes Study [8], 40% of the cohort of 1015 patients was prescribed clozapine. Given the limited efficacy of current antipsychotics, it is likely that increasing numbers of patients may be treated with clozapine. Despite advantages for some patients, clinicians need to be aware of the propensity of this drug to cause a wide range of adverse reactions, some of which are quite rare. While 10 cases of acute interstitial nephritis associated with clozapine have been reported to the MHRA, far fewer cases appear to have occurred with most other antipsychotics [6]. For example, only one case has been reported for risperidone and haloperidol [6], antipsychotics that have been widely prescribed. As far as the authors are aware, there are no reports of acute interstitial nephritis caused by other atypical antipsychotics although there are reports of acute renal failure occurring as a consequence of neuroleptic malignant syndrome.

## Conclusions

The purpose of this case report is to highlight to clinicians the rare potential of clozapine to cause acute renal failure and the importance of early recognition and treatment. As adverse renal events occur rarely, there is a paucity of advice for psychiatrists but the Maudsley Prescribing Guidelines have a brief but useful section on the use of antipsychotics in people with renal impairment and helpful guidance on the use of clozapine in general [9]. Clinicians initiating clozapine need to be aware of the possibility of a drug induced renal adverse event and it is recommended that baseline renal function is always assessed before clozapine treatment is started, and monitored during the initiation phase if necessary. Patients, like the one reported here, who have had a previous trial of clozapine that was discontinued due to a suspected adverse drug reaction, should only be rechallenged with clozapine with due caution in case some form of sensitization has developed. Psychiatrists and psychiatric nurses should be watchful for the following presenting features that may suggest a possible drug induced acute interstitial nephritis: acute onset of renal failure, usually within days of exposure to clozapine; fever and tachycardia; skin rash; eosinophilia, elevated IgE and CRP. Early involvement of a renal physician is recommended if such a reaction is suspected.

## Consent

Written informed consent was obtained from the patient for publication of this case report and accompanying images. A copy of the written consent is available for review by the Editor-in-Chief of this journal.

## Competing interests

The authors have no competing interests.

## Authors' contributions

RH was the psychiatrist attending the patient and who took responsibility for the writing of this report; TG provided pharmacy expertise; FQ attended the patient during the clinical incident described and DM and nursing colleagues provided expert care for the patient throughout. SS provided valuable additional pharmacy and research support for the write-up.
